# Adverse childhood and adult experiences, intrinsic capacity decline, and subsequent physical-psychological-cognitive multimorbidity: a prospective cohort study from China

**DOI:** 10.1016/j.jnha.2026.100832

**Published:** 2026-03-28

**Authors:** Keke Liu, Huijuan Luo, Boran E, Huining Kuang, Chenyu Zhang, Xin Guo

**Affiliations:** a School of Public Health, Capital Medical University, Beijing, People's Republic of China; b School Health Center, Beijing Center for Disease Prevention and Control, Beijing, 100069, People's Republic of China; c Chinese Center for Disease Control and Prevention, Beijing, People's Republic of China

**Keywords:** Intrinsic capacity, Life-course adversity, Multimorbidity, Aging, China, CHARLS

## Abstract

**Background:**

Life-course adversities are important determinants of unhealthy aging. Intrinsic capacity (IC) may capture early functional vulnerability before disease onset, but evidence linking adverse childhood and adulthood experiences (ACEs and AAEs) to IC and later physical-psychological-cognitive multimorbidity (PPC-MM) remains limited. This study aimed to examine whether ACEs and AAEs are associated with intrinsic capacity and subsequent PPC-MM in a nationally representative cohort.

**Methods:**

We analyzed 2,559 middle-aged and older adults from the China Health and Retirement Longitudinal Study who were free of multimorbidity at baseline. ACEs and AAEs were assessed retrospectively. IC was constructed across cognitive, locomotor, psychological, sensory, and vitality domains and treated as the primary outcome. PPC-MM was examined as a secondary outcome using Cox regression and multistate models.

**Results:**

Higher cumulative ACE exposure was significantly associated with lower IC (*β* = −0.04 per additional ACE; 95% CI −0.07 to −0.00), whereas AAEs showed no significant association with IC. Among specific adversities, childhood physical abuse was strongly associated with reduced IC (*β* = −0.12; 95% CI −0.22 to −0.02). Both ACEs and AAEs were independently associated with increased risks of PPC-MM. Each additional ACE increased the hazard of PPC-MM by 19% (HR 1.19; 95% CI 1.06–1.33), while each additional AAE increased risk by 18% (HR 1.18; 95% CI 1.03–1.34). Individuals exposed to both ACEs and AAEs exhibited the highest risk of physical–psychological multimorbidity (HR 1.77; 95% CI 1.28–2.46). Multistate analyses showed that multimorbidity accumulated progressively, most commonly transitioning from physical–psychological combinations to PPC-MM.

**Conclusion:**

Childhood adversity is associated with reduced intrinsic capacity in later life, reflecting early functional vulnerability, while both childhood and adulthood adversities contribute to downstream multimorbidity. These findings support IC as an early, adversity-sensitive marker linking life-course stress to multimorbidity and highlight opportunities for upstream intervention to promote healthy aging.

## 1 Introduction

The long-term health consequences of life-course adversities, particularly adverse childhood experiences (ACEs) and adverse adulthood experiences (AAEs), have been well-documented [[1], [2], [3]]. Substantial evidence indicates that ACEs (e.g., abuse, neglect, and household dysfunction) and AAEs (e.g., economic hardship, major illness) are strongly associated with a range of chronic physical diseases, mental health issues, and cognitive decline [[3], [4], [5], [6], [7], [8], [9]]. Notably, the health impact of ACEs often exhibits a dose-response relationship, and early adversities may increase the likelihood of encountering stressors in adulthood, creating a cumulative burden [[10], [11], [12], [13], [14]]. However, most prior work has focused on static disease counts or single clinical outcomes, often overlooking the functional deterioration that precedes the accumulation of diagnosed chronic conditions.

Intrinsic capacity (IC), proposed by the World Health Organization as the composite of an individual’ s physical and mental capacities at any point in time, has gained increasing attention as a function-centered framework for healthy aging [15,16]. The IC construct encompasses five domains-cognition, locomotion, psychological capacity, sensory function, and vitality—and is designed to capture early, subclinical changes in functional reserve [16]. Importantly, declines in IC have been consistently associated with higher risks of hospitalization and all-cause mortality, as well as increased incidence of major geriatric syndromes, including falls, frailty, sarcopenia, urinary incontinence, and functional disability, across diverse populations and clinical settings [[17], [18], [19]]. Because IC captures functional vulnerability before disease onset, it serves as an integrative, early-warning indicator that may be more sensitive to life-course adversities than multimorbidity alone.

Downstream from this functional decline is physical-psychological-cognitive multimorbidity (PPC-MM), defined as the co-occurrence of conditions affecting physical, mental, and/or cognitive health, represents one of the most prevalent and disabling multimorbidity patterns among middle-aged and older adults [20]. Evidence from the Global Ageing, Health, and Policy initiatives, including cohorts from 32 countries (e.g., the UK, USA, China, and India), indicates that the prevalence of PPC-MM ranges between 8.1% and 33.9% [20] In China, the proportion of middle-aged and older adults with at least two chronic conditions has reached 56.7%, underscoring the growing burden of multimorbidity in the world's largest aging population [21]. Due to its multidimensional impact, PPC-MM severely compromises quality of life and poses substantial clinical and socioeconomic challenges to healthcare systems worldwide [20,22]. Rather than an abrupt clinical event, the development of PPC-MM is a dynamic, multi-stage process. Understanding this progression requires examining transitions between health states rather than deterministic outcomes.

Despite accumulating evidence, few studies have explicitly positioned IC as the primary outcome in relation to life-course adversity, nor have they integrated the joint effects of childhood and adulthood adversities to map the transition from functional decline to complex multimorbidity. To address these gaps, this prospective cohort study uses data from the China Health and Retirement Longitudinal Study (CHARLS). We examined how ACEs and AAEs are associated with (1) intrinsic capacity in later life as an early functional marker, and (2) subsequent transitions to physical-psychological-cognitive multimorbidity, including intermediate multimorbidity states. In doing so, we aim to advance a life-course framework for early prevention and targeted intervention in aging populations.

## 2 Methods

### 2.1 Study design and participants

This study utilized data from the CHARLS, a nationally representative cohort survey that includes adults aged 45 years and older from approximately 150 counties across 28 provinces in China. The baseline survey was conducted in 2011, followed by follow-up waves in 2013, 2015, and 2018. In addition, a life history survey conducted in 2014 collected retrospective information on participants’ life-course experiences from birth.

The initial sample for this study was derived from the harmonized CHARLS dataset (2011–2018), which included 25,586 respondents. Through a 1:1 match with the 2014 Life History Survey data, we identified 20,252 participants with complete longitudinal records. Subsequently, the following exclusion criteria were applied sequentially: (1) 7,320 individuals with incomplete longitudinal follow-up information; (2) 363 individuals aged below 45 years at baseline; (3) 5,721 participants with missing data on key outcome variables, including chronic diseases, depressive symptoms, cognitive function and IC; (4) 3,201 individuals with incomplete exposure information on adverse childhood experiences or adverse adult experiences; and (5) 1,088 individuals who already had PPC-MM at at the 2011 baseline survey. After these exclusions, the final analytical sample consisted of 2,559 participants (see Figure S1).

It should be noted that while multimorbidity outcomes were prospectively ascertained through 2018, IC was assessed using data only from the 2011, 2013, and 2015 survey waves. This limitation arose because the 2018 wave lacked several essential objective IC indicators—particularly in the locomotion and vitality domains, such as gait speed (or components of the Short Physical Performance Battery, SPPB), handgrip strength, and lung function. Since these were structurally absent rather than sporadically missing, all IC-related analyses were confined to the 2011–2015 period to ensure measurement validity and longitudinal comparability. The CHARLS protocol was approved by the Biomedical Ethics Review Committee of Peking University (IRB00001052-11015), and written informed consent was obtained from all participants.

### 2.2 Adverse childhood and adulthood experiences

This study selected ten widely used indicators of ACEs, which were categorized into three domains: household dysfunction (comprising household substance use, household mental illness, domestic violence, household criminal behavior, parental separation/divorce, and parental death), neglect and abuse (including physical neglect, emotional neglect, and physical abuse), and other ACEs (exposure to natural disasters) [4]. All ACE indicators were treated as binary variables, with specific definitions provided in Table S1. Five items measuring AAEs were adapted from previous studies [23], including the death of a child, lifetime experience of discrimination, long-term bed rest, long-term hospitalization, and job loss due to health reasons. All items were also dichotomously coded, as detailed in Table S1. Based on participants' exposure to adversities across childhood and adulthood, they were classified into four groups: a none group (no experience of adversities in either childhood or adulthood), an ACEs-only group (experienced childhood adversities but no adulthood adversities), an AAEs-only group (experienced adulthood adversities but no childhood adversities), and an ACEs and AAEs group (experienced adversities in both childhood and adulthood).

### 2.3 Intrinsic capacity

Based on the intrinsic capacity framework outlined in the World Health Organization's Integrated Care for Older People guidelines, and incorporating evidence from systematic reviews and cohort studies [15,[24], [25], [26]], we developed a composite IC score ranging from 0 to 10. The IC construct encompassed five domains: locomotor, cognitive, sensory, vitality, and psychological. (1) Locomotor capacity was evaluated using the SPPB, which assesses balance, gait speed, and chair stand performance. (2) Cognitive function was measured with a modified version of the Telephone Interview for Cognitive Status (TICS), adapted from the U.S. Health and Retirement Study. This instrument evaluated both crystallized cognition (numerical ability, orientation, visuospatial skills) and episodic memory (immediate and delayed recall). (3) Sensory function was determined through self-reported hearing and vision impairments. (4) Vitality was operationalized using two objective indicators: pulmonary function (peak expiratory flow) and handgrip strength. (5) Psychological status was assessed with the 10-item Center for Epidemiologic Studies Depression Scale (CESD-10). Each IC domain was classified into three levels—severely impaired, mildly impaired, or optimal—and assigned a score from 0 to 2. The overall IC score was calculated by summing the five domain-specific scores, with higher values indicating better intrinsic capacity. This composite scoring approach is consistent with previous epidemiological studies and reflects the WHO framework of intrinsic capacity as a multidimensional functional construct [27]. Detailed definitions of each domain and scoring procedures are provided in the Supplementary Material (Table S1).

### 2.4 Physical, psychological, and cognitive multimorbidity

Building on the intrinsic capacity assessment described in Section 2.3, PPC-MM was conceptualized as a clinical health outcome reflecting the co-occurrence of physical, psychological, and cognitive conditions. Physical, psychological, and cognitive health statuses were assessed at baseline and follow-up to quantify this progression. Chronic diseases, including hypertension, diabetes, cancer, and 11 other conditions (14 in total), were identified based on affirmative responses to the question: "Have you been diagnosed with these diseases by a doctor?" The total number of diseases was calculated (range: 0–14). Depressive symptoms were measured using the CES-D-10, with a total score ≥10 indicating clinically significant depressive symptoms. Cognitive function was evaluated independently across three domains: episodic memory (immediate and delayed word recall), orientation (through tests of temporal, spatial, and personal orientation), and executive function (serial 7 subtraction test). Within each wave, mean scores were computed for each cognitive domain [28]. Participants were divided into age groups: Group 1 (45–49), Group 2 (50–54), Group 3 (55–59), Group 4 (60–64), Group 5 (65–69), Group 6 (70–74), Group 7 (75–79), and Group 8 (≥80). Cognitive impairment was defined as scoring more than 1.5 standard deviations below the age-specific mean in at least one cognitive domain [29]. Based on these definitions, the study focused on four incident PPC-MM patterns: physical-psychological multimorbidity, physical-cognitive multimorbidity, psychological-cognitive multimorbidity, and physical-psychological-cognitive multimorbidity.

### 2.5 Covariates

Demographic information included age, sex (male or female), residential area (rural or urban), marital status (married or other), and educational level (primary, secondary, and higher education). Health behavior factors included smoking status, drinking status (categorized as never or former/current), and physical activity. Participants who engaged in physical activity at least three times per week were classified as physically active; otherwise, they were classified as inactive.

### 2.6 Statistical analysis

Baseline characteristics were summarized as means (standard deviations) for continuous variables and frequencies (percentages) for categorical variables. Group comparisons were conducted according to exposure to ACEs and AAEs at baseline using one-way analysis of variance for continuous variables and chi-square tests for categorical variables. To assess potential selection bias, an attrition analysis was conducted using chi-square and t-tests to compare baseline characteristics between participants included in the final analytical cohort and those excluded during the sample selection process. Due to 897 missing values in the physical activity variable, multiple imputation by chained equations was employed to generate 10 imputed datasets after incorporating baseline characteristics. Primary analyses were repeated across all imputed datasets and pooled according to Rubin's rules. The primary outcome was IC, treated as a continuous variable and analyzed using multivariable linear regression models. Multimorbidity outcomes were analyzed as secondary outcomes. Multimorbidity analyses were conducted to examine whether life-course adversities associated with reduced intrinsic capacity were also related to subsequent disease accumulation. Regression coefficients (*β*) and 95% confidence intervals (CIs) were reported to quantify mean differences in IC scores associated with ACEs, AAEs, and their joint exposure. For each exposure-outcome combination, three models were sequentially fitted: Model 1 was unadjusted; Model 2 adjusted for sociodemographic covariates (age, sex, marital status, educational level, and residential area); Model 3 further adjusted for health behaviors (physical activity, smoking status, and drinking status) in addition to Model 2 covariates. Covariate selection was based on previous evidence concerning social and behavioral determinants of multimorbidity and the long-term effects of early-life adversity [30].

To explore heterogeneity across functional domains, secondary analyses examined associations of specific types of ACEs and AAEs (e.g., domestic violence, household mental illness, parental separation or divorce, experiencing lifetime discrimination, and ever being hospitalized for a month or longer) with both the overall IC score (using linear regression) and incident PPC-MM (using Cox models). The same covariate adjustment strategy (Model 3) was applied. To assess whether life-course adversities translated into downstream disease accumulation, Cox proportional hazards regression models were used to estimate associations of cumulative ACEs, AAEs, and their temporal combinations with incident PPC-MM and its component patterns, reporting hazard ratios (HRs) and 95% CIs. Cumulative incidence function (CIF) curves were constructed to visualize PPC-MM risk over time across adversity exposure groups, with model adjustment following the same sequential strategy described above.

Subgroup analyses were conducted stratified by sex, age group (<65 vs ≥65 years), marital status, and residential area using fully adjusted models. The higher education subgroup was not analyzed separately due to limited sample size (n = 68). Sensitivity analyses included (1) mutual adjustment for ACEs and AAEs and (2) models examining individual ACE or AAE indicators instead of cumulative scores. Finally, an exploratory multi-state model was fitted to describe transitions between physical, psychological, and cognitive morbidity states. These analyses were descriptive and hypothesis-generating. All statistical analyses were performed using R version 4.5.1 and Stata version 17.0, with statistical significance set at *P* < 0.05.

## 3 Results

### 3.1 Cohort characteristics and adversity exposure patterns

The final analytical cohort comprised 2,559 participants free of multimorbidity at baseline. As presented in Table 1, significant demographic and socioeconomic gradients were observed across life-course adversity exposure categories. At baseline, mean intrinsic capacity scores were similar across life-course adversity exposure categories (*P* = 0.357), supporting the prospective design for examining adversity as a predictor of IC decline. Participants with combined adverse childhood and adulthood experiences constituted the most vulnerable subgroup, characterized by an older mean age (58.5 vs. 56.5 years, *P* < 0.001), the highest male predominance (64.9% vs. 39.7%), the greatest proportion with only primary education (56.3% vs. 39.7%), and the highest current smoking prevalence (37.7% vs. 22.2%). When examined separately (Table S2), both ACEs and AAEs were significantly associated with lower socioeconomic status and adverse health behaviors. Correlation analyses revealed predominantly weak but significant interrelationships among most adversity indicators (Tables S3–S5), suggesting a clustering of disadvantage across the life course. Compared with individuals excluded from the analysis, participants included in the analytical cohort were slightly younger (mean age 57.4 vs. 59.1 years), and were more likely to be male (59.7% vs. 45.9%), urban residents (43.9% vs. 39.9%), married (93.6% vs. 86.0%), and had higher educational attainment (secondary or higher: 48.8% vs. 27.8%) (Table S6).

**Table 1:** Baseline characteristics of study participants by life-course adversity exposure patterns (N = 2,559).

		Exposure to adverse childhood and adulthood experiences across life-course				
Characteristics	Total	None	ACEs only	AAEs only	ACEs and AAEs	*P* value
Intrinsic capacity score, mean (SD)	7.7 (1.1)	7.8 (1.1)	7.7 (1.1)	7.9 (1.1)	7.7 (1.2)	0.357
Age, mean (SD), y	57.4 (7.8)	56.51 (8.4)	57.0 (7.6)	57.7 (7.6)	58.5 (7.8)	<0.001
Sex, n (%)						
Male	1,527 (59.7)	50 (39.7)	988 (59.2)	13 (40.6)	476 (64.9)	0.005
Female	1,032 (40.3)	76 (60.3)	680 (40.8)	19 (59.4)	257 (35.1)	
Residential area, n (%)						
Rural	1,435 (56.1)	54 (42.9)	952 (57.1)	13 (40.6)	416 (56.8)	0.005
Urban	1,124 (43.9)	72 (57.1)	716 (42.9)	19 (59.4)	317 (43.2)	
Marital status, n (%)						
Married	2,396 (93.6)	115 (91.3)	1,571 (94.2)	31 (96.9)	679 (92.6)	0.271
Other marital status	163 (6.4)	11 (8.7)	97 (5.8)	1 (3.1)	54 (7.4)	
Educational level, n (%)						
Primary education	1,310 (51.2)	50 (39.7)	832 (49.9)	15 (46.9)	413 (56.3)	0.004
Secondary education	1,232 (48.1)	75 (59.5)	826 (49.5)	16 (50.0)	315 (43.0)	
Higher education	17 (0.7)	1 (0.8)	10 (0.6)	1 (3.1)	5 (0.7)	
Smoking status, n (%)						
Non-smoker	1,668 (65.2)	98 (77.8)	1,091 (65.4)	22 (68.8)	457 (62.3)	0.009
Current smoker	891 (34.8)	28 (22.2)	577 (34.6)	10 (31.3)	276 (37.7)	
Drinking status, n (%)						
Non-drinker	1,521 (59.4)	90 (71.4)	983 (58.9)	22 (68.8)	426 (58.1)	0.061
Current drinker	1,038 (40.6)	36 (28.6)	685 (41.1)	10 (31.3)	307 (41.9)	
Physical activity (≥3 × a week), n (%)						
Yes	2,493 (97.4)	125 (99.2)	1,619 (97.1)	32 (100.0)	717 (97.8)	0.259
No	66 (2.6)	1 (0.8)	49 (2.9)	0 (0.0)	16 (2.2)	

Abbreviation: ACEs = Adverse Childhood Experiences; AAEs = Adverse Adulthood Experiences.

### 3.2 Life-course adversities and intrinsic capacity

As the primary outcome of this study, intrinsic capacity was first examined in relation to life-course adversities. Table 2 presents the associations between life-course adversities and intrinsic capacity score. In the fully adjusted model, a higher number of ACEs, modeled as a continuous variable, was significantly associated with a lower intrinsic capacity score (*β* = −0.04, 95% CI: −0.07 to −0.00). Similar estimates were observed in the crude and sociodemographic-adjusted models. When ACEs were categorized, participants reporting two or more ACEs showed a lower intrinsic capacity score compared with those reporting no ACEs (*β* = −0.11, 95% CI: −0.29 to 0.07 in the fully adjusted model), although the confidence interval included the null. A consistent dose–response pattern was observed across categories.

**Table 2:** Associations of life-course adversity with intrinsic capacity score at baseline: results from crude and adjusted linear regression models (n = 2,559).

Exposure to ACEs and AAEs	N	Intrinsic Capacity score		
		Model 1	Model 2	Model 3
		*B* (95%CI)	*B* (95%CI)	*B* (95%CI)
Total	2,559			
ACEs				
0	154	1.0 Ref	1.0 Ref	1.0 Ref
1	387	−0.05 (−0.26, 0.15)	−0.07 (−0.27, 0.14)	−0.06 (−0.27, 0.14)
≥2	2,018	−0.15 (−0.33, 0.03)	−0.11 (−0.29, 0.07)	−0.11 (−0.29, 0.07)
ACEs (1-indicator per increasing)a		−0.05 (−0.08, −0.01)	−0.04 (−0.07, −0.00)	−0.04 (−0.07, −0.00)
AAEs				
0	1,794	1.0 Ref	1.0 Ref	1.0 Ref
1	436	−0.07 (−0.18, 0.05)	−0.03 (−0.14, 0.09)	−0.02 (−0.14, 0.09)
≥2	329	−0.01 (−0.14, 0.12)	0.02 (−0.11, 0.15)	0.02 (−0.11, 0.15)
AAEs (1-indicator per increasing)a		−0.00 (−0.05, 0.05)	0.01 (−0.03, 0.06)	0.01 (−0.03, 0.06)
Life-course Adversity				
None	123	1.0 Ref	1.0 Ref	1.0 Ref
ACEs only	1,671	−0.10 (−0.30, 0.11)	−0.07 (−0.27, 0.13)	−0.07 (−0.27, 0.13)
AAEs only	31	0.11 (−0.32, 0.55)	0.14 (−0.29, 0.57)	0.15 (−0.28, 0.58)
ACEs and AAEs	734	−0.14 (−0.36, 0.07)	−0.08 (−0.29, 0.13)	−0.08 (−0.29, 0.13)

Abbreviation: ACEs, adverse childhood experiences; AAEs, adverse adulthood experiences; HR, hazard ratio; 95% CI, 95% confidence interval; Ref, Reference.

β indicates the mean difference in intrinsic capacity score compared with the reference group a Continuous variable. Model 1: the crude model. Model 2: adjusted for age, sex, residential area, marital status and educational level.

Model 3: adjusted for age, sex, residential area, marital status, educational level, smoking, drinking and physical activity.

In contrast, AAEs, whether modeled as continuous or categorical variables, were not significantly associated with intrinsic capacity in any model. The estimates for both one and two or more AAEs were close to null, with confidence intervals crossing zero. Overall, these findings indicate that childhood adversities, rather than adulthood adversities, are more consistently associated with reduced intrinsic capacity in later life.

### 3.3 Specific adversities and intrinsic capacity

**Fig. 1:**
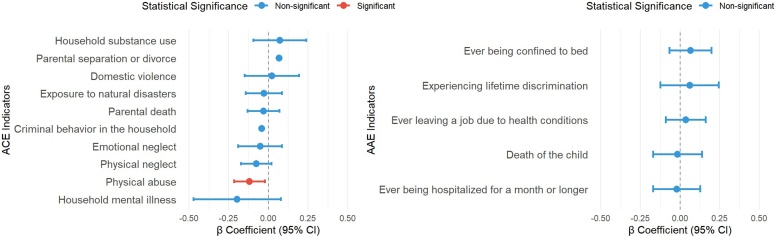
Association Analysis Between Specific Adversity Indicators and Total Intrinsic Capacity Score. (A) Association Between Specific Childhood Adversities and Intrinsic Capacity. (B) Association Between Specific Adverse Adulthood Experiences and Intrinsic Capacity. Abbreviation: *β*, Regression coefficients; 95% CI, 95% confidence interval.

Fig. 1 shows the associations between specific adverse experiences and intrinsic capacity score. Among childhood adversities, physical abuse was significantly associated with a lower intrinsic capacity score (*β* = −0.12, 95% CI: −0.22 to −0.02). Other childhood adversities, including parental mental illness, emotional neglect, parental death, and exposure to natural disasters, were not significantly associated with intrinsic capacity.

No significant associations were observed between individual adulthood adversities and intrinsic capacity. Estimates for adulthood adversities, such as prolonged hospitalization, unemployment due to health reasons, discrimination experiences, and being bedridden, were generally small and imprecise, with confidence intervals including the null. For several low-prevalence adversities, wide confidence intervals were observed, reflecting limited statistical power.

These results suggest that the adverse impact of childhood adversity on intrinsic capacity may be driven by specific early-life stressors, whereas adulthood adversities appear less strongly related to baseline functional capacity. Given these findings on intrinsic capacity, we next examined whether life-course adversities were associated with subsequent risks of physical, psychological, and cognitive multimorbidity.

### 3.4 Life-course adversities and physical-psychological-cognitive multimorbidity

After examining intrinsic capacity as the primary outcome, we next assessed whether life-course adversities were associated with multimorbidity trajectories. Cox regression analyses demonstrated consistent dose-response relationships between adversity exposure and multimorbidity risk (Table 3). When modeled as continuous variables, each additional ACE was associated with significantly increased hazards of physical–psychological (HR 1.15, 95% confidence interval [CI] 1.10–1.21), physical–cognitive (HR 1.08, 95% CI 1.01–1.15), psychological–cognitive (HR 1.14, 95% CI 1.03–1.26), and Physical–psychological–cognitive multimorbidity (HR 1.19, 95% CI 1.06–1.33). Similarly, each additional AAE independently elevated risks across all multimorbidity patterns, with effect sizes ranging from HR = 1.11 (95% CI, 1.03–1.20) for physical–cognitive to HR = 1.18 (95% CI, 1.03–1.34) for Physical–psychological–cognitive multimorbidity.

**Table 3:** Associations of life-course adversity with the risk of different multimorbidity patterns at follow-up (n = 2,559).

Exposure to ACEs and AAEs	N	Physical–psychological multimorbidity	Physical–cognitive multimorbidity	Psychological–cognitive multimorbidity	Physical–psychological–cognitive multimorbidity
		Model^b^	Model^b^	Model^b^	Model^b^
		HR(95%CI)	HR(95%CI)	HR(95%CI)	HR(95%CI)
Total	2,559				
ACEs					
0	154	1.0 Ref	1.0 Ref	1.0 Ref	1.0 Ref
1	387	1.26 (0.92−1.73)	0.85 (0.58−1.24)	0.91 (0.52−1.62)	1.23 (0.58−2.61)
≥2	2,018	1.51 (1.14−2.01)	0.95 (0.69−1.32)	0.98 (0.60−1.62)	1.41 (0.72−2.77)
ACEs (1-indicator per increasing)^a^		1.15 (1.10−1.21)	1.08 (1.01−1.15)	1.14 (1.03−1.26)	1.19 (1.06−1.33)
AAEs					
0	1,794	1.0 Ref	1.0 Ref	1.0 Ref	1.0 Ref
1	436	1.17 (1.01−1.36)	0.90 (0.73−1.12)	0.81 (0.58−1.14)	0.84 (0.57−1.24)
≥2	329	1.48 (1.27−1.74)	1.25 (1.01−1.54)	1.41 (1.03−1.94)	1.42 (0.99−2.05)
AAEs (1-indicator per increasing)^a^		1.17 (1.11−1.24)	1.11 (1.03−1.20)	1.15 (1.02−1.29)	1.18 (1.03−1.34)
Life-course Adversity					
None	123	1.0 Ref	1.0 Ref	1.0 Ref	1.0 Ref
ACEs only	1,671	1.36 (0.99−1.88)	1.06 (0.71−1.56)	0.82 (0.48−1.40)	1.34 (0.62−2.87)
AAEs only	31	1.05 (0.54−2.04)	1.65 (0.83−3.25)	0.45 (0.10−1.96)	0.97 (0.20−4.68)
ACEs and AAEs	734	1.77 (1.28−2.46)	1.08 (0.72−1.62)	0.90 (0.52−1.57)	1.44 (0.66−3.16)

Abbreviation: ACEs, adverse childhood experiences; AAEs, adverse adulthood experiences; HR, hazard ratio; 95% CI, 95% confidence interval; Ref, Reference.

a Continuous variable.

b Model adjusted for age, sex, residential area, marital status, educational level, smoking, drinking and physical activity.

Categorical analysis revealed a cumulative risk pattern, with the strongest association observed for physical–psychological multimorbidity among those dually exposed to ACEs and AAEs (HR 1.77, 95% CI 1.28–2.46). The persistence of these associations in sensitivity analyses employing different model specifications (Table S7) confirmed the robustness of our findings.

### 3.5 Specific adversities and physical-psychological-cognitive multimorbidity

**Fig. 2:**
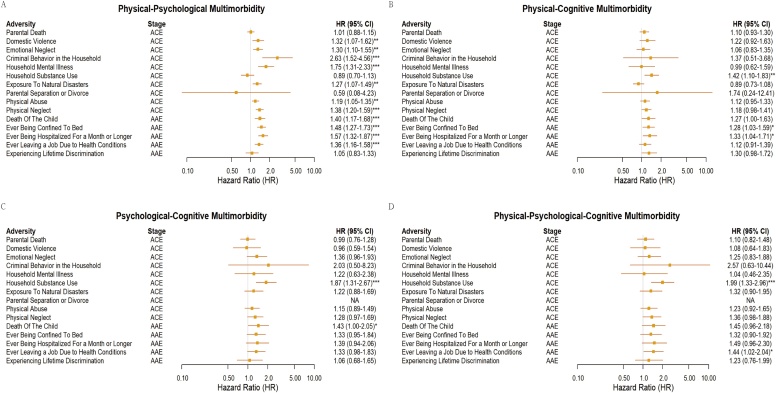
Hazard ratios for associations of each indicator of adverse childhood experiences (ACEs) and adverse adulthood experiences (AAEs) with Multimorbidity Risk. (A). Physical–Psychological Multimorbidity. (B). Physical–Cognitive Multimorbidity. (C). Psychological–Cognitive Multimorbidity. (D). Physical–Psychological–Cognitive Multimorbidity.

Mutual adjustment analyses confirmed the independent contributions of both ACEs and AAEs to multimorbidity risk (Table S9). Examination of specific adversities revealed distinct risk signatures across multimorbidity types (Fig. 2). Household criminal behavior (HR 2.63, 95% CI 1.52–4.56), parental mental illness (HR 1.75, 95% CI 1.31–2.33), and physical neglect (HR 1.38, 95% CI 1.20–1.59) emerged as potent childhood risk factors for physical–psychological multimorbidity, while adulthood health adversities including prolonged hospitalization (HR 1.57, 95% CI 1.32–1.87) and bed confinement (HR 1.48, 95% CI 1.27–1.73) represented strong adult predictors. Household substance abuse demonstrated particularly broad effects, significantly increasing risks for physical–cognitive (HR 1.42, 95% CI 1.10–1.83), psychological–cognitive (HR 1.87, 95% CI 1.31–2.67), and Physical–psychological–cognitive multimorbidity (HR 1.99, 95% CI 1.33–2.96).

### 3.6 Temporal patterns and heterogeneity in risk associations in relation to intrinsic capacity and multimorbidity

Cumulative incidence analyses demonstrated a clear, dose-dependent increase in multimorbidity risk associated with adversity exposure across all multimorbidity patterns (Figure S2). Participants with ≥2 ACEs or ≥2 AAEs consistently exhibited the steepest incidence trajectories, with the highest cumulative risks observed among individuals exposed to both childhood and adulthood adversities. These divergence patterns widened progressively over the follow-up period, indicating an accelerating accumulation of multimorbidity risk over time. Importantly, when interpreted alongside the intrinsic capacity findings, these temporal patterns are consistent with the possibility that early functional vulnerability may precede the development of multimorbidity. Childhood adversities, particularly physical abuse, were associated with lower intrinsic capacity at baseline. This association reflects a compromised functional reserve that exists even before the onset of multimorbidity. The steeper cumulative incidence curves observed among individuals with high adversity exposure may therefore partly reflect a life-course process in which may contribute to increased susceptibility to later multimorbidity development.

Stratified analyses further revealed substantial heterogeneity across demographic subgroups (Table S8). The associations between life-course adversity and multimorbidity were generally stronger among women, married individuals, rural residents, and younger adults (<65 years), although patterns varied by multimorbidity type. These subgroup differences may also reflect differential levels of intrinsic capacity resilience, as women and rural residents are more likely to experience compounded social disadvantage and limited access to health-promoting resources across the life course. Notably, combined exposure to ACEs and AAEs showed the most robust and consistent associations across subgroups, particularly for physical–psychological multimorbidity among older adults (HR 2.05, 95% CI 1.11–3.78) and rural residents (HR 1.92, 95% CI 1.15–3.20). This pattern is consistent with a cumulative stress model, whereby early-life erosion of intrinsic capacity interacts with subsequent adult adversities to accelerate functional decline and disease clustering.

### 3.7 Health State transitions and dynamic patterns of multimorbidity in the context of functional decline

Figure S3A illustrates the overall transitions among different health states during follow-up. Among 2,559 participants free of PPC-MM at baseline, 26.3% developed physical–psychological multimorbidity, 13.9% progressed to physical–cognitive multimorbidity, and 2.3% to psychological–cognitive multimorbidity. Notably, 4.8% of those with initial two-domain multimorbidity further evolved into physical–psychological–cognitive multimorbidity during subsequent waves, indicating a cumulative deterioration trend. When integrated with intrinsic capacity results, these transition patterns suggest that multimorbidity accumulation is not a random process but follows a functionally structured trajectory. Participants exposed to childhood adversity exhibited lower intrinsic capacity at baseline, which may be related to earlier transitions from a healthy state to two-domain multimorbidity, particularly involving physical and psychological domains.

Figure S3B presents the complete transition pathways among participants initially healthy (n = 807). The majority of new cases emerged from the physical–psychological domain (6.3%), followed by physical–cognitive (4.5%) and psychological–cognitive (2.0%) combinations. A small subset (1.4%) directly transitioned to the three-domain PPC-MM state, suggesting that multimorbidity accumulation commonly proceeds through intermediate two-domain stages. This stepwise transition pattern is consistent with a conceptual framework in which declines in intrinsic capacity represent an early, subclinical phase of vulnerability that precedes overt disease clustering. In this context, intrinsic capacity may act as a functional integrator linking life-course adversity to downstream multimorbidity trajectories, particularly for combinations involving psychological and cognitive domains. Taken together, these multistate patterns visually confirm the progressive and hierarchical accumulation of multimorbidity across physical, psychological, and cognitive dimensions.

## 4 Discussion

In this nationally representative longitudinal study, we examined how life-course adversities relate to functional and clinical aging trajectories. Adverse childhood experiences were associated with lower IC in later life among individuals free of multimorbidity at baseline, suggesting that early-life adversity may manifest as reduced functional reserve before overt disease onset. In contrast, adverse adulthood experiences showed weaker associations with IC but stronger associations with subsequent PPC-MM. Multistate analyses indicated that multimorbidity commonly developed through intermediate two-domain stages rather than abrupt clustering. Together, these findings position intrinsic capacity as an early adversity-sensitive functional indicator and multimorbidity as a later clinical manifestation within a life-course framework of aging.

The most robust and clinically meaningful finding concerns the association between adverse childhood experiences and intrinsic capacity, the primary outcome of this study. Higher ACE scores were associated with lower IC in a dose–response manner across crude and fully adjusted models, even among individuals without multimorbidity at baseline. This suggests that IC captures a subclinical, integrative dimension of health vulnerability that is not reducible to diagnosed disease. Among specific adversities, physical abuse emerged as the strongest predictor of reduced IC, whereas other childhood adversities showed weaker or non-significant associations. Severe early-life stressors involving direct physical threat may exert particularly durable effects on physiological and psychological reserve. Prior studies indicate that childhood maltreatment induces long-term dysregulation of the hypothalamic–pituitary–adrenal (HPA) axis, chronic low-grade inflammation, and neurodevelopmental alterations, which together compromise vitality, cognition, and emotional regulation [[31], [32], [33]]. Epigenetic modifications, telomere shortening, and structural brain changes further support the biological embedding of early stress [[34], [35], [36], [37]]. In contrast, the absence of significant associations between adulthood adversity and IC underscores the developmental sensitivity of intrinsic capacity to early-life exposures, consistent with the life course perspective and cumulative inequality theory [38].

While IC was the primary outcome, the multimorbidity analyses provide important secondary evidence that early functional impairment may translate into accelerated disease accumulation over time. Both ACEs and AAEs were associated with increased risks of PPC-MM in a dose-response manner. Notably, the association for AAEs was stronger, which aligns with life course theory, suggesting that health outcomes are often more strongly associated with proximal risk factors [[39], [40], [41]].

Participants exposed to childhood adversity already exhibited lower intrinsic capacity at baseline, which may partially explain their steeper cumulative incidence of multimorbidity and earlier transitions into disease states. The multistate analyses revealed that multimorbidity accumulation most often proceeded through intermediate two-domain combinations, rather than an abrupt transition to PPC-MM. Such patterns are consistent with a functional-to-clinical progression model, in which reduced intrinsic capacity lowers resilience and increases susceptibility to multiple co-occurring conditions [42,43]. Importantly, the differential associations of ACEs with IC and AAEs with multimorbidity suggest temporal complementarity rather than redundancy. Childhood adversity lays the foundation for long-term functional vulnerability, while adulthood adversity acts as a proximal trigger, accelerating the clinical manifestation of disease [[44], [45], [46], [47]].

This study further reveals that different types of adversity exert heterogeneous effects on functional decline and multimorbidity outcomes. This study revealed that severe family dysfunction, including household criminal behavior, parental mental illness, and physical neglect, showed the strongest associations with physical–psychological multimorbidity. In contrast, household substance use demonstrated broad effects across multiple multimorbidity domains, particularly for combinations involving cognition, a pattern that may be attributable to its association with accelerated aging and the consequent increased risk of cognitive impairment [48].

In adulthood, health-related adversities such as ever being confined to bed and ever being hospitalized for a month or longer were strongly associated with multimorbidity but not intrinsic capacity, reflecting the direct impact of physiological system damage on multimorbidity onset, with long-term physical inactivity leading to significant declines in both physical and cognitive function [49]. Psychosocial stressors such as bereavement were particularly associated with psychological-cognitive multimorbidity, consistent with prior evidence linking severe stress to prolonged psychological and cognitive dysfunction [50]. Together, these findings reinforce the conceptual distinction between adversity-related functional vulnerability, captured by intrinsic capacity, and clinically manifest multimorbidity, while highlighting the heterogeneity of biological and behavioral pathways across adversity types.

Subgroup analyses further revealed that women and rural residents exhibited stronger adversity-multimorbidity associations. These patterns may reflect differential distributions of intrinsic capacity and recovery resources across populations. Rural residents often face compounded social disadvantage and limited access to preventive care [51], while women are often characterized by greater cumulative adversity exposure, heightened health vigilance, and more proactive care-seeking across the life course [[52], [53], [54]]. Such heterogeneity underscores the importance of IC-informed risk stratification in identifying populations most vulnerable to downstream multimorbidity.

By identifying intrinsic capacity as an early, adversity-sensitive functional outcome, this study highlights opportunities for upstream intervention before multimorbidity becomes established. Routine assessment of IC in midlife and early older age, particularly among individuals with known childhood adversity, may facilitate early identification of functional decline and targeted prevention strategies. At the clinical and public health levels, interventions aimed at preserving or restoring intrinsic capacity (e.g., physical activity, cognitive training, psychosocial support) may help buffer the long-term health consequences of early-life adversity and delay multimorbidity onset [15,55]. These findings support a shift from disease-centered models toward function-centered approaches in life-course healthy aging.

This study has several strengths. First, it utilized data from a large, nationally representative longitudinal cohort with repeated health assessments across physical, psychological, and cognitive domains. Second, by positioning intrinsic capacity as the primary outcome and multimorbidity as a secondary outcome, this study offers a novel functional perspective on adversity-related aging trajectories. Third, the use of multi-state models provided insights into the dynamic and temporal nature of multimorbidity transitions, which are seldom explored in population-based studies.

However, several limitations should be considered. First, adverse childhood and adulthood experiences were assessed retrospectively using self-reported life-history data and may be subject to recall bias. In addition, some potentially relevant adversities were not captured in the CHARLS life-history survey, such as exposure to neighborhood violence, school bullying, or certain occupational hazards. The analytical sample also differed from excluded participants in several sociodemographic characteristics, which may introduce potential selection bias. Second, although intrinsic capacity and multimorbidity were examined within a shared life-course framework, this study did not formally test mediation pathways between functional decline and disease accumulation. Third, limited sample sizes for some multimorbidity transition pathways may have reduced statistical power for rare transitions in the multistate analyses. Fourth, the observational design precludes causal inference. Finally, although the CHARLS cohort is nationally representative of middle-aged and older adults in China, the generalizability of these findings to other cultural and healthcare contexts requires further investigation.

## 5 Conclusion

In conclusion, this study demonstrates that childhood adversity is associated with reduced intrinsic capacity in later life, while both childhood and adulthood adversities are associated with increased risks of physical, psychological, and cognitive multimorbidity. Intrinsic capacity may represent an early, integrative marker of vulnerability that precedes the development of multimorbidity within a life-course framework of aging. These findings highlight the importance of incorporating life-course adversity and functional capacity assessment into strategies for healthy aging and chronic disease prevention.

## CRediT authorship contribution statement

Keke Liu: Writing – original draft, Formal analysis, Data curation, Conceptualization. Huijuan Luo: Formal analysis, Data curation. Boran E: Supervision, Methodology, Conceptualization. Huining Kuang: Validation, Formal analysis, Data curation. Chenyu Zhang: Formal analysis, Software. Xin Guo: Writing – review & editing, Supervision, Funding acquisition, Conceptualization.

## Consent for publication

Not applicable.

## Funding sources

Supported by the Ministry of Science and Technology of the People’s Republic of China, National Key Research and Development Program of China (2021YFC2702102), the Beijing Municipal Health Commission High-level Public Health Technical Talent Construction Project (Leading Talent-01-09), and the Operation of Public Health Emergency Response Mechanism (102393220020010000017).

## Ethics approval and consent to participate

This study is a secondary analysis of the data from the CHARLS. Ethics approval was obtained from the Biomedical Ethics Review Board of Peking University in China (IRB00001052–11015).

## Declaration of Generative AI and AI-assisted technologies in the writing process

AI was not used in the preparation of this manuscript nor its table or figures.

## Data availability statement

The database was used from the China Health and Retirement Longitudinal Study (http://charls.pku.edu.cn/).

## Declaration of competing interest

The authors declare that they have no known competing financial interests or personal relationships that could have appeared to influence the work reported in this paper.

## Glossary

PPC-MM: Physical-psychological-cognitive multimorbidity
IC: intrinsic capacity
ACEs: Adverse childhood experiences
AAEs: Adverse adulthood experiences
CHARLS: China Health and Retirement Longitudinal Study
SPPB: Short Physical Performance Battery
TICS: Telephone Interview for Cognitive Status
CESD-10: 10-item Center for Epidemiologic Studies Depression Scale
*β*: Regression coefficients
CIF: Cumulative incidence function
HRs: hazard ratios
CI: Confidence interval
HPA: Hypothalamic-pituitary-adrenal
