# Supplementary material for: Adverse childhood and adult experiences, intrinsic capacity decline, and subsequent physical-psychological-cognitive multimorbidity: a prospective cohort study from China
Source: J Nutr Health Aging. 2026 Mar 28;30(5):100832. doi: 10.1016/j.jnha.2026.100832 (PMC13049300; doi:10.1016/j.jnha.2026.100832)
Supplement: Supplementary file 1 [file mmc1.docx]

| **Table S1.** Question items and responses for variables included in the adverse childhood and adulthood experiences. | | |
| --- | --- | --- |
| **Categories** | | **Questionnaire items** |
| **Adverse childhood experiences (ACEs)** | | |
| Household dysfunction | Household substance use | During the years you were growing up, did your female/male guardian ever have alcoholism or drug? (Responses were categorized as 0=no and 1=yes). |
|  | Household mental illness | Did your female/male guardian have an abnormality of mind when you were young? (Responses were categorized as 0=no and 1=yes). |
|  | Domestic violence | Have your father/mother ever beat up your mother/father? (Responses were categorized to 0=not very often or never; 1=often or sometimes). |
|  | Criminal behavior in the household | During the years you were growing up, have your female/male guardian ever been involved in criminal activities like burglary or selling stolen property, or arrested or sent to prison? (Responses were categorized as 0=no and 1=yes). |
|  | Parental separation or divorce | Were your biological parents divorced (including a long separation due to emotional problems) before you were 17 years old? |
|  | Parental death | Either of the parents was dead before the participant was 17 years old. (The answer was calculated based on the date of birth and their parental death, and responses were categorized to 0=no and 1=yes). |
| Neglect and abuse | Physical neglect | When you were a child before age 17, was there ever a time when your family did not have enough food to eat? (Responses were categorized as 0=no and 1=yes). |
|  | Emotional neglect | How much love and affection did your female guardian give you while you were growing up? (Responses were categorized as 0=often or sometimes; 1=rarely or never). |
|  | Physical abuse | When you were growing up, did your female/male guardian ever hit you? (Responses were categorized as 0=not very often or never; 1=often or sometimes). |
| Additional ACE | Exposure to natural disasters | Between 1958-1962 did you and your family (including your grandparents, parents, siblings, children and so on) experience starvation or move away from the famine-stricken area? (Responses were categorized as 0=no and 1=yes). |
| **Adverse adulthood experiences (AAEs)** | | |
|  | Death of the child | Death of the participant’s child (Responses were categorized as 0=no and 1=yes). |
|  | Experiencing lifetime discrimination | After you were 16 years old, because of ill health, did you experience any of the following (denied promotions, assignment to a task with fewer responsibilities, working on tasks below your qualifications, harassment by your boss or colleagues, pay cuts, dismissed)? (Responses were categorized as 0=no and 1=yes). |
|  | Ever being confined to bed | After you were 16 years old, because of a health condition, were you ever confined to bed or home for one month or more? (Responses were categorized as 0=no and 1=yes). |
|  | Ever being hospitalized for a month or longer | After you were 16 years old, because of a health condition, were you ever hospitalized for a month or more? (Responses were categorized as 0=no and 1=yes). |
|  | Ever leaving a job due to health conditions | After you were 16 years old, because of a health condition, did you leave your job for one month or more? (Responses were categorized as 0=no and 1=yes). |
| **Intrinsic capacity ( the IC score for this study was set as the sum of the scores (0-10) of the five dimensions)** | | |
| Locomotion | | This dimension was measured with reference to the SPBB scale and included assessments of walking speed, balance and chair rise. Participants who walked 4 meters in less than 4.82 seconds, 4.82-6.20 seconds, 6.21-8.70 seconds, more than 8.70 seconds, and could not complete the walk were assigned a score of 4 to 0, respectively, in descending order. Participants who could stand with feet together and half-foot spacing for 10 seconds were assigned a score of 1, those who did not hold it for 10 seconds and did not attempt it were assigned a score of 0. Holding a full-foot spacing for 10 seconds was assigned a score of 2, holding it for 3-9.9 seconds was assigned a score of 1, and those who held it for <3 seconds and did not attempt it were assigned a score of 0. The time for the 5 chair rises was categorized as <11.19 seconds, 11.2-13.69 seconds, 13.7-16.69 seconds, 16.7-59.9 seconds, >60 seconds or those who could not complete it were assigned a value of 4-0 points in descending order, respectively. The SPBB score was the sum of the three, and the motor dimension was divided into three levels according to the SPBB score, which was 0 (0≤SPBB≤2), 1 (3≤SPBB≤9), and 2 (10≤SPBB≤12), respectively. |
| Sensory | | The sensory dimension consists of two parts: vision and hearing. The two entries in the vision section are “Would you say your eyesight for seeing things at a distance is excellent, very good, good, fair, or poor?” and “Would you say your eyesight for seeing things up close is excellent, very good, good, fair, or poor?” Participants who self-reported excellent, very good, good were scored as 1, fair was scored as 0.5, and poor and participants who wore glasses or were blind were scored 0. The entry for the listening section was “Would you say your hearing is excellent, very good, good, fair, or poor?” The scoring rules for hearing are the same as for vision. |
| Vitality | | The vitality dimension consists of two dimensions: lung capacity and grip strength. The three spirometry measurements were taken as the maximum value and scored 0 and 1 for males and females based on the threshold value of 350 L/min and 220 L/min, respectively. Grip strength was taken as the maximum of the dominant hand measurements and was scored 0 and 1 for males and females according to the threshold values of 28 kg and 18 kg, respectively. |
| Psychological | | The 10-item version of the Centre for Epidemiological Studies Depression scale (CESD-10) has 10 items for assessing depressive states. It contains 10 items, each with 4 options for the past week: little or no time (< 1 day); not much (1-2 days); sometimes or half of the time (3-4 days); most or all (5-7 days). The total scores range between 0 and 30, with higher scores indicating higher levels of depressive symptoms. The psychological dimensions were assigned a score of 0 (20≤CESD≤30), 1 (10≤CESD<20), and 2 (0≤CESD<10), respectively. |
| Cognitive | | The cognitive assessment tools used in CHARLS include intelligence level and situational memory. Intelligence was assessed by subtracting 7 (5 consecutive times) from 100, answering the current year, month, day, day of the week, and season, and visuospatial ability was assessed by drawing two overlapping pentagons, with scores ranging from 0-11; situational memory was assessed by the number of immediate and delayed recalls of 10 Chinese words, with scores ranging from 0-10. Each of the two dimensions was scored as 0 if any dimension was below one standard deviation, and the others were scored as 1. The cognitive dimension was scored as the sum of the two dimensions (0-2). |
| **Physical, psychological and cognitive conditions** | | |
| Physical condition | | The respondent reported having hypertension, diabetes, cancer, lung disease, heart problem, stroke, psychiatricproblems, arthritis, dyslipidemia, liver disease, kidney disease, stomach or digestivedisease, asthma and/or memory-related disease (Responses were categorized as 0=no and 1=yes). |
| Psychological condition | | CESD-10 score ≥10 (Responses were categorized as 0=no and 1=yes). |
| Cognitive conditions | | Having the score ≥1.5 standard deviations below age group-specific means in at least one of four cognitive domains: immediate word recall, delayed word recall, serial 7’s subtraction test, and orientation (Responses were categorized as 0=no and 1=yes). |

Abbreviation: ACEs, adverse childhood experiences; AAEs, adverse adulthood experiences; IC, Intrinsic capacity.

| **Table S2.** Baseline characteristics of participants by adverse childhood experiences (ACEs) and adverse adulthood experiences (AAEs) (N=2,559). | | | | | | |
| --- | --- | --- | --- | --- | --- | --- |
| **Characteristics** | **Adverse childhood experiences (ACEs)** | | | **Adverse adulthood experiences (AAEs)** | | |
|  | **No** | **Yes** | ***P*** value | **No** | **Yes** | ***P*** value |
| **Age, mean (SD), y** | 56.3 ± 8.2 | 57.7 ± 7.7 | 0.009 | 57.1 ± 7.6 | 58.7 ± 7.9 | <0.001 |
| **Sex, n (%)** |  |  |  |  |  |  |
| Male | 63 (39.9) | 1,464 (61.0) | <0.001 | 1,038 (57.9) | 489 (63.9) | 0.004 |
| Female | 95 (60.1) | 937 (39.0) |  | 756 (42.1) | 276 (36.1) |  |
| **Residential area, n (%)** |  |  |  |  |  |  |
| Rural | 67 (42.4) | 1,368 (57.0) | <0.001 | 1,006 (56.1) | 429 (56.1) | 0.517 |
| Urban | 91 (57.6) | 1,033 (43.0) |  | 788 (43.9) | 336 (43.9) |  |
| **Marital status, n (%)** |  |  |  |  |  |  |
| Married | 146 (92.4) | 2,250 (93.7) | 0.515 | 1,686 (94.0) | 710 (92.8) | 0.267 |
| Other marital status | 12 (7.6) | 151 (6.3) |  | 108 (6.0) | 55 (7.2) |  |
| **Educational level, n (%)** |  |  |  |  |  |  |
| Primary education | 65 (41.1) | 1,245 (51.9) | 0.025 | 882 (49.2) | 428 (55.9) | 0.005 |
| Secondary education | 91 (57.6) | 1,141 (47.5) |  | 901 (50.2) | 331 (43.3) |  |
| Higher education | 2 (1.3) | 15 (0.6) |  | 11 (0.6) | 6 (0.8) |  |
| **Smoking status, n (%)** |  |  |  |  |  |  |
| Non-smoker | 120 (75.9) | 1,548 (64.5) | 0.003 | 1,189 (66.3) | 479 (62.6) | 0.075 |
| Current smoker | 38 (24.1) | 853 (35.5) |  | 605 (33.7) | 286 (37.4) |  |
| **Drinking status, n (%)** |  |  |  |  |  |  |
| Non-drinker | 112 (70.9) | 1,409 (58.7) | 0.002 | 1,073 (59.8) | 448 (58.6) | 0.556 |
| Current drinker | 46 (29.1) | 992 (41.3) |  | 721 (40.2) | 317 (41.4) |  |
| **Physical activity (≥ 3 × a week), n (%)** |  |  |  |  |  |  |
| Yes | 157 (99.4) | 2,336 (97.3) | 0.111 | 1,744 (97.2) | 749 (97.9) | 0.310 |
| No | 1 (0.6) | 65 (2.7) |  | 50 (2.8) | 16 (2.1) |  |

| **Table S3.** Phi correlation coefficients between adverse childhood experiences (ACEs). | | | | | | | | | | |
| --- | --- | --- | --- | --- | --- | --- | --- | --- | --- | --- |
| **Variable** | **Household substance use** | **Household mental illness** | **Domestic violence** | **Criminal behavior in the household** | **Parental separation or divorce** | **Parental death** | **Physical neglect** | **Emotional neglect** | **Physical abuse** | **Exposure to natural disasters** |
| Household substance use | 1 |  |  |  |  |  |  |  |  |  |
| Household mental illness | 0.014 | 1 |  |  |  |  |  |  |  |  |
| Domestic violence | 0.052** | 0.057** | 1 |  |  |  |  |  |  |  |
| Criminal behavior in the household | .023* | 0.024* | 0.069** | 1 |  |  |  |  |  |  |
| Parental separation or divorce | -0.012 | -0.007 | -0.012 | -0.003 | 1 |  |  |  |  |  |
| Parental death | 0 | 0.009 | -0.001 | 0.019 | -0.027** | 1 |  |  |  |  |
| Physical neglect | 0.030** | 0.041** | 0.043** | 0.021* | 0.009 | 0.065** | 1 |  |  |  |
| Emotional neglect | 0.006 | -0.002 | 0.106** | 0.009 | 0.012 | -0.034** | 0.057** | 1 |  |  |
| Physical abuse | 0.001 | 0.013 | 0.166** | 0.030** | .032** | -0.016 | 0.013 | 0.579** | 1 |  |
| Exposure to natural disasters | 0.009 | 0.002 | 0.029** | -0.025* | -0.026** | 0.004 | 0.427** | 0.044** | 0.027** | 1 |
| **P* < 0.05, ** *P* < 0.01 | | | | | | | | | | |

| **Table S4.** Phi correlation coefficients between adverse adulthood experiences (AAEs). | | | | | |
| --- | --- | --- | --- | --- | --- |
| **Variable** | **Death of the child** | **Experiencing lifetime discrimination** | **Ever being confined to bed** | **Ever being hospitalized for a month or longer** | **Ever leaving a job due to health conditions** |
| Death of the child | 1 |  |  |  |  |
| Experiencing lifetime discrimination | .045** | 1 |  |  |  |
| Ever being confined to bed | 0.011 | 0.136** | 1 |  |  |
| Ever being hospitalized for a month or longer | 0.006 | 0.122** | 0.489** | 1 |  |
| Ever leaving a job due to health conditions | 0.013 | 0.232** | 0.488** | 0.453** | 1 |
| **P* < 0.05, ** *P* < 0.01 | | | | | |

| **Table S5.** Phi correlation coefficients between adverse adulthood experiences (AAEs) and adverse childhood experiences (ACEs). | | | | | | | | | | |
| --- | --- | --- | --- | --- | --- | --- | --- | --- | --- | --- |
| **Variable** | **Household substance use** | **Household mental illness** | **Domestic violence** | **Criminal behavior in the household** | **Parental separation or divorce** | **Parental death** | **Physical neglect** | **Emotional neglect** | **Physical abuse** | **Exposure to natural disasters** |
| Death of the child | 0.036** | 0.012 | 0.037** | 0.036** | 0.017 | 0.038** | 0.042** | 0.033** | 0.036** | 0.031** |
| Experiencing lifetime discrimination | 0.001 | 0.013 | .019* | 0.006 | -0.011 | -0.008 | 0.011 | 0.027** | 0.036** | 0.001 |
| Ever being confined to bed | -0.002 | 0.025* | 0.043** | 0.007 | -0.016 | 0.048** | 0.038** | -0.012 | 0.006 | 0.030** |
| Ever being hospitalized for a month or longer | -.024* | .075** | 0.019 | 0.014 | -0.014 | 0.029** | 0.025* | 0.014 | 0.001 | 0.01 |
| Ever leaving a job due to health conditions | -0.013 | 0.017 | 0.012 | 0.020* | -0.018 | 0.021* | 0.072** | 0.039** | 0.039** | 0.045** |
| **P* < 0.05, ** *P* < 0.01 | | | | | | | | | | |

**Table S6**. Baseline characteristics of the included analytical sample versus the excluded participants.

| **Characteristics** | | | | | | **Included (n=2,559)** | | | | | | **Excluded (n=17,693)** | | | | | | **P-value** | | |  |
| --- | --- | --- | --- | --- | --- | --- | --- | --- | --- | --- | --- | --- | --- | --- | --- | --- | --- | --- | --- | --- | --- |
| **Age, mean (SD), y** | | | | | | 57.4 (7.8) | | | | | | 59.1 (10.5) | | | | | | <0.001 | | |  |
| **Sex, n (%)** | | | | | |  | | | | | |  | | | | | | <0.001 | | |  |
| Male | | | | | | 1,527 (59.7) | | | | | | 8,123 (45.9) | | | | | |  | | |  |
| Female | | | | | | 1,032 (40.3) | | | | | | 9,570 (54.1) | | | | | |  | | |  |
|  | | | | | |  | | | | | |  | | | | | |  | | |  |
| **Residential area, n (%)** | | | | | |  | | | | | |  | | | | | | <0.001 | | |  |
| Rural | | | | | | 1,435 (56.1) | | | | | | 10,634 (60.1) | | | | | |  | | |  |
| Urban | | | | | | 1,124 (43.9) | | | | | | 7,059 (39.9) | | | | | |  | | |  |
|  | | | | | |  | | | | | |  | | | | | |  | | |  |
| **Marital status, n (%)** | | | | | |  | | | | | |  | | | | | | <0.001 | | |  |
| Married | | | | | | 2,396 (93.6) | | | | | | 15,216 (86.0) | | | | | |  | | |  |
| Other marital status | | | | | | 163 (6.4) | | | | | | 2,442 (13.8) | | | | | |  | | |  |
|  | | | | | |  | | | | | |  | | | | | |  | | |  |
| **Educational level, n (%)** | | | | | |  | | | | | |  | | | | | | <0.001 | | |  |
| Primary education | | | | | | 1,310 (51.2) | | | | | | 12,704 (71.8) | | | | | |  | | |  |
| Secondary education | | | | | | 1,232 (48.1) | | | | | | 4,777 (27.0) | | | | | |  | | |  |
| Higher education | | | | | | 17 (0.7) | | | | | | 142 (0.8) | | | | | |  | | |  |
| **Table S7**. Associations of life-course adversity with the risk of different multimorbidity patterns at follow-up: results from Crude and Adjusted Models (n=2,559). | | | | | | | | | | | | | | | | | | | | | |
| **Exposure to ACEs and AAEs** | **N** | | **Physical–psychological multimorbidity** | | | | | **Physical–cognitive multimorbidity** | | | | | **Psychological–cognitive multimorbidity** | | | | **Physical–psychological–cognitive multimorbidity** | | | | |
|  |  |  | **Model 1** | | **Model 2** | | | **Model 1** | | **Model 2** | | | **Model 1** | | **Model 2** | | **Model 1** | | | **Model 2** | |
|  |  |  | **HR(95%CI)** | | **HR(95%CI)** | | | **HR(95%CI)** | | **HR(95%CI)** | | | **HR(95%CI)** | | **HR(95%CI)** | | **HR(95%CI)** | | | **HR(95%CI)** | |
| **Total** | 2,559 | |  | |  | | |  | |  | | |  | |  | |  | | |  | |
| **ACEs** |  | |  | |  | | |  | |  | | |  | |  | |  | | |  | |
| 0 | 154 | | 1.0 Ref | | 1.0 Ref | | | 1.0 Ref | | 1.0 Ref | | | 1.0 Ref | | 1.0 Ref | | 1.0 Ref | | | 1.0 Ref | |
| 1 | 387 | | 1.21 (0.88-1.67) | | 1.28 (0.93-1.75) | | | 0.90 (0.61-1.31) | | 0.87 (0.60-1.27) | | | 0.94 (0.53-1.65) | | 0.95 (0.54-1.68) | | 1.29 (0.61-2.72) | | | 1.28 (0.61-2.71) | |
| ≥2 | 2,018 | | 1.47 (1.11-1.95) | | 1.52 (1.14-2.02) | | | 1.05 (0.76-1.45) | | 0.96 (0.69-1.33) | | | 1.00 (0.61-1.64) | | 1.01 (0.61-1.66) | | 1.49 (0.76-2.91) | | | 1.44 (0.73-2.83) | |
| ACEs (1-indicator per increasing)a |  | | 1.13 (1.08-1.18) | | 1.15 (1.09-1.20) | | | 1.09 (1.02-1.16) | | 1.07 (1.01-1.15) | | | 1.12 (1.02-1.23) | | 1.14 (1.03-1.25) | | 1.18 (1.05-1.31) | | | 1.18 (1.06-1.32) | |
| **AAEs** |  | |  | |  | | |  | |  | | |  | |  | |  | | |  | |
| 0 | 1,794 | | 1.0 Ref | | 1.0 Ref | | | 1.0 Ref | | 1.0 Ref | | | 1.0 Ref | | 1.0 Ref | | 1.0 Ref | | | 1.0 Ref | |
| 1 | 436 | | 1.22 (1.05-1.42) | | 1.17 (1.01-1.36) | | | 0.98 (0.79-1.21) | | 0.90 (0.73-1.12) | | | 0.89 (0.64-1.25) | | 0.82 (0.58-1.15) | | 0.93 (0.63-1.36) | | | 0.85 (0.58-1.25) | |
| ≥2 | 329 | | 1.48 (1.27-1.74) | | 1.49 (1.27-1.74) | | | 1.28 (1.04-1.59) | | 1.25 (1.01-1.55) | | | 1.39 (1.01-1.91) | | 1.42 (1.03-1.95) | | 1.43 (0.99-2.05) | | | 1.43 (1.00-2.06) | |
| AAEs (1-indicator per increasing)a |  | | 1.17 (1.11-1.24) | | 1.17 (1.11-1.24) | | | 1.12 (1.04-1.21) | | 1.11 (1.03-1.20) | | | 1.15 (1.02-1.29) | | 1.15 (1.03-1.29) | | 1.18 (1.04-1.34) | | | 1.18 (1.04-1.34) | |
| **Life-course Adversity** |  | |  | |  | | |  | |  | | |  | |  | |  | | |  | |
| None | 123 | | 1.0 Ref | | 1.0 Ref | | | 1.0 Ref | | 1.0 Ref | | | 1.0 Ref | | 1.0 Ref | | 1.0 Ref | | | 1.0 Ref | |
| ACEs only | 1,671 | | 1.32 (0.96-1.82) | | 1.37 (0.99-1.88) | | | 1.16 (0.79-1.71) | | 1.07 (0.72-1.58) | | | 0.86 (0.51-1.46) | | 0.85 (0.50-1.45) | | 1.44 (0.67-3.08) | | | 1.38 (0.64-1.38) | |
| AAEs only | 31 | | 1.10 (0.56-2.14) | | 1.04 (0.53-2.03) | | | 1.81 (0.92-3.57) | | 1.65 (0.83-3.25) | | | 0.53 (0.12-2.30) | | 0.47 (0.11-2.04) | | 1.14 (0.24-5.47) | | | 0.99 (0.21-0.99) | |
| ACEs and AAEs | 734 | | 1.76 (1.27-2.44) | | 1.78 (1.28-2.47) | | | 1.25 (0.84-1.87) | | 1.09 (0.73-1.64) | | | 0.98 (0.57-1.70) | | 0.94 (0.54-1.63) | | 1.63 (0.75-3.55) | | | 1.49 (0.68-1.49) | |
| Abbreviation: ACEs, adverse childhood experiences; AAEs, adverse adulthood experiences; HR, hazard ratio; 95% CI, 95% confidence interval; Ref, Reference. a Continuous variable.  Model 1: the crude model. Model 2: adjusted for age, sex, residential area, marital status and educational level. | | | | | | | | | | | | | | | | | | | | | |
| **Table S8.** Subgroup analysis: the association between life-course adversity and physical, psychological and cognitive multimorbidity (n=2559). | | | | | | | | | | | | | | | | | | | | | |
| **Exposure to ACEs and AAEs** | | **Age, stratified HR (95% CI)** | | | | | **Sex, stratified HR (95% CI)** | | | | **Marry, stratified HR (95% CI)** | | | | | **Residence, stratified HR (95% CI)** | | | | | |
|  |  | **<65 years** | | **≥65 years** | | | **Men** | | **Women** | | **Other** | | | **Married** | | **Urban** | | | **Rural** | | |
| **Physical, psychological multimorbidity** | | | | | | | | | | | | | | | | | | | | | |
| **ACEs** | |  | |  | | |  | |  | |  | | |  | |  | | |  | | |
| 0 | | 1.0 Ref | | 1.0 Ref | | | 1.0 Ref | | 1.0 Ref | | 1.0 Ref | | | 1.0 Ref | | 1.0 Ref | | | 1.0 Ref | | |
| 1 | | 1.33 (0.91-1.95) | | 1.17 (0.66-2.09) | | | 1.06 (0.63-1.80) | | 1.40 (0.94-2.08) | | 0.94 (0.39-2.25) | | | 1.29 (0.92-1.82) | | 1.08 (0.71-1.67) | | | 1.51 (0.93-2.45) | | |
| ≥2 | | 1.41 (1.00-1.99) | | 1.67 (1.01-2.78) | | | 1.35 (0.84-2.16) | | 1.59 (1.11-2.27) | | 1.60 (0.78-3.30) | | | 1.50 (1.10-2.04) | | 1.30 (0.90-1.88) | | | 1.81 (1.15-2.83) | | |
| ACEs (1-indicator per increasing)^a^ | | 1.15 (1.08-1.22) | | 1.14 (1.05-1.24) | | | 1.14 (1.07-1.22) | | 1.15 (1.08-1.24) | | 1.32 (1.13-1.53) | | | 1.14 (1.08-1.19) | | 1.11 (1.04-1.20) | | | 1.18 (1.11-1.25) | | |
| **AAEs** | |  | |  | | |  | |  | |  | | |  | |  | | |  | | |
| 0 | | 1.0 Ref | | 1.0 Ref | | | 1.0 Ref | | 1.0 Ref | | 1.0 Ref | | | 1.0 Ref | | 1.0 Ref | | | 1.0 Ref | | |
| 1 | | 1.14 (0.93-1.40) | | 1.23 (0.98-1.55) | | | 1.19 (0.96-1.47) | | 1.14 (0.92-1.42) | | 1.12 (0.72-1.74) | | | 1.18 (1.01-1.39) | | 1.39 (1.10-1.76) | | | 1.04 (0.85-1.27) | | |
| ≥2 | | 1.59 (1.30-1.94) | | 1.35 (1.04-1.75) | | | 1.46 (1.19-1.80) | | 1.49 (1.17-1.89) | | 1.14 (0.67-1.92) | | | 1.53 (1.29-1.80) | | 1.55 (1.20-1.99) | | | 1.44 (1.17-1.76) | | |
| AAEs (1-indicator per increasing)^a^ | | 1.19 (1.10-1.28) | | 1.15 (1.05-1.26) | | | 1.16 (1.07-1.25) | | 1.18 (1.08-1.29) | | 1.09 (0.90-1.33) | | | 1.18 (1.11-1.25)** | | 1.19 (1.09-1.30) | | | 1.16 (1.08-1.24) | | |
| **Life-course Adversity** | |  | |  | | |  | |  | |  | | |  | |  | | |  | | |
| None | | 1.0 Ref | | 1.0 Ref | | | 1.0 Ref | | 1.0 Ref | | 1.0 Ref | | | 1.0 Ref | | 1.0 Ref | | | 1.0 Ref | | |
| ACEs only | | 1.24 (0.85-1.81) | | 1.62 (0.88-2.98) | | | 1.14 (0.68-1.92) | | 1.48 (0.98-2.23) | | 1.89 (0.76-4.69) | | | 1.28 (0.91-1.81) | | 1.16 (0.76-1.76) | | | 1.60 (0.97-2.64) | | |
| AAEs only | | 0.83 (0.35-2.01) | | 1.48 (0.51-4.26) | | | 0.81 (0.23-2.81) | | 1.11 (0.50-2.47) | | 2.60 (0.61-11.09) | | | 0.83 (0.38-1.79) | | 1.20 (0.52-2.80) | | | 0.84 (0.28-2.53) | | |
| ACEs and AAEs | | 1.67 (1.13-2.46) | | 2.05 (1.11-3.78) | | | 1.51 (0.89-2.55) | | 1.87 (1.23-2.86) | | 1.99 (0.78-5.05) | | | 1.72 (1.21-2.44) | | 1.69 (1.10-2.61) | | | 1.92 (1.15-3.20) | | |
| **Physical, cognitive multimorbidity** | | | | | | | | | | | | | | | | | | | | | |
| **ACEs** | |  | |  | | |  | |  | |  | | |  | |  | | |  | | |
| 0 | | 1.0 Ref | | 1.0 Ref | | | 1.0 Ref | | 1.0 Ref | | 1.0 Ref | | | 1.0 Ref | | 1.0 Ref | | | 1.0 Ref | | |
| 1 | | 1.09 (0.66-1.80) | | 0.57 (0.31-1.03) | | | 0.66 (0.36-1.22) | | 0.99 (0.61-1.61) | | 0.56 (0.21-1.52) | | | 0.89 (0.59-1.35) | | 0.95 (0.51-1.77) | | | 0.78 (0.48-1.26) | | |
| ≥2 | | 1.21 (0.77-1.89) | | 0.61 (0.37-1.00) | | | 0.83 (0.49-1.40) | | 1.02 (0.67-1.55) | | 1.04 (0.50-2.17) | | | 0.93 (0.65-1.35) | | 0.98 (0.57-1.67) | | | 0.90 (0.59-1.37) | | |
| ACEs (1-indicator per increasing)^a^ | | 1.11 (1.02-1.20) | | 1.00 (0.90-1.12) | | | 1.06 (0.97-1.16) | | 1.09 (1.00-1.20) | | 1.31 (1.09-1.57) | | | 1.05 (0.98-1.12) | | 1.07 (0.95-1.19) | | | 1.08 (1.00-1.16) | | |
| **AAEs** | |  | |  | | |  | |  | |  | | |  | |  | | |  | | |
| 0 | | 1.0 Ref | | 1.0 Ref | | | 1.0 Ref | | 1.0 Ref | | 1.0 Ref | | | 1.0 Ref | | 1.0 Ref | | | 1.0 Ref | | |
| 1 | | 0.88 (0.67-1.18) | | 0.93 (0.67-1.28) | | | 0.87 (0.64-1.18) | | 0.93 (0.69-1.26) | | 0.59 (0.32-1.10) | | | 0.96 (0.77-1.21) | | 0.89 (0.61-1.31) | | | 0.91 (0.70-1.17) | | |
| ≥2 | | 1.40 (1.07-1.83) | | 1.06 (0.74-1.52) | | | 1.32 (1.00-1.73) | | 1.14 (0.81-1.61) | | 0.45 (0.19-1.06) | | | 1.38 (1.10-1.72) | | 1.00 (0.66-1.52) | | | 1.36 (1.06-1.75) | | |
| AAEs (1-indicator per increasing)^a^ | | 1.15 (1.05-1.27) | | 1.05 (0.92-1.19) | | | 1.10 (1.00-1.22) | | 1.12 (0.99-1.26) | | 0.79 (0.58-1.08) | | | 1.14 (1.05-1.24) | | 1.03 (0.89-1.20) | | | 1.14 (1.04-1.25) | | |
| **Life-course Adversity** | |  | |  | | |  | |  | |  | | |  | |  | | |  | | |
| None | | 1.0 Ref | | 1.0 Ref | | | 1.0 Ref | | 1.0 Ref | | 1.0 Ref | | | 1.0 Ref | | 1.0 Ref | | | 1.0 Ref | | |
| ACEs only | | 1.51 (0.86-2.63) | | 0.60 (0.34-1.05) | | | 0.88 (0.48-1.62) | | 1.17 (0.70-1.96) | | 1.10 (0.50-2.42) | | | 1.09 (0.69-1.71) | | 0.91 (0.51-1.62) | | | 1.16 (0.68-2.00) | | |
| AAEs only | | 2.40 (0.99-5.80) | | 0.97 (0.32-2.97) | | | 1.61 (0.51-5.12) | | 1.64 (0.70-3.85) | | 0.71 (0.09-5.91) | | | 1.95 (0.93-4.08) | | 0.62 (0.14-2.76) | | | 2.55 (1.13-5.75) | | |
| ACEs and AAEs | | 1.59 (0.89-2.83) | | 0.60 (0.34-1.07) | | | 0.94 (0.50-1.75) | | 1.14 (0.66-1.95) | | 0.58 (0.24-1.40) | | | 1.20 (0.76-1.92) | | 0.87 (0.47-1.60) | | | 1.22 (0.70-2.12) | | |
| **Psychological, cognitive multimorbidity** | | | | | | | | | | | | | | | | | | | | | |
| **ACEs** | | 1.0 Ref | | 1.0 Ref | | | 1.0 Ref | | 1.0 Ref | | 1.0 Ref | | | 1.0 Ref | | 1.0 Ref | | | 1.0 Ref | | |
| 0 | | 1.07 (0.55-2.08) | | 0.62 (0.20-1.90) | | | 0.49 (0.21-1.16) | | 1.28 (0.60-2.74) | | 0.25 (0.06-1.01) | | | 1.18 (0.60-2.31) | | 1.16 (0.31-4.38) | | | 0.84 (0.44-1.57) | | |
| 1 | | 1.00 (0.55-1.81) | | 0.93 (0.37-2.34) | | | 0.50 (0.24-1.03) | | 1.47 (0.74-2.90) | | 0.88 (0.37-2.10) | | | 1.06 (0.57-1.96) | | 1.95 (0.61-6.27) | | | 0.77 (0.44-1.34) | | |
| ≥2 | | 1.13 (1.00-1.27) | | 1.15 (0.96-1.37) | | | 1.05 (0.90-1.21) | | 1.22 (1.07-1.39) | | 1.30 (1.02-1.66) | | | 1.11 (1.00-1.24) | | 1.21 (1.00-1.46) | | | 1.11 (0.99-1.24) | | |
| ACEs (1-indicator per increasing)^a^ | |  | |  | | |  | |  | |  | | |  | |  | | |  | | |
| **AAEs** | |  | |  | | |  | |  | |  | | |  | |  | | |  | | |
| 0 | | 1.0 Ref | | 1.0 Ref | | | 1.0 Ref | | 1.0 Ref | | 1.0 Ref | | | 1.0 Ref | | 1.0 Ref | | | 1.0 Ref | | |
| 1 | | 0.70 (0.44-1.11) | | 0.97 (0.58-1.61) | | | 0.98 (0.60-1.61) | | 0.70 (0.44-1.11) | | 0.32 (0.11-0.88) | | | 0.97 (0.68-1.39) | | 1.02 (0.54-1.94) | | | 0.74 (0.50-1.11) | | |
| ≥2 | | 1.70 (1.16-2.47) | | 1.03 (0.57-1.89) | | | 1.53 (0.98-2.39) | | 1.34 (0.85-2.12) | | 0.64 (0.23-1.82) | | | 1.62 (1.15-2.27) | | 1.61 (0.85-3.06) | | | 1.38 (0.96-1.99) | | |
| AAEs (1-indicator per increasing)^a^ | | 1.22 (1.06-1.40) | | 1.05 (0.85-1.29) | | | 1.20 (1.02-1.40) | | 1.10 (0.92-1.31) | | 0.74 (0.47-1.16) | | | 1.21 (1.07-1.36) | | 1.23 (0.98-1.55) | | | 1.13 (0.99-1.29) | | |
| **Life-course Adversity** | |  | |  | | |  | |  | |  | | |  | |  | | |  | | |
| None | | 1.0 Ref | | 1.0 Ref | | | 1.0 Ref | | 1.0 Ref | | 1.0 Ref | | | 1.0 Ref | | 1.0 Ref | | | 1.0 Ref | | |
| ACEs only | | 0.82 (0.44-1.54) | | 0.83 (0.30-2.32) | | | 0.42 (0.19-0.93) | | 1.23 (0.60-2.54) | | 0.69 (0.29-1.67) | | | 0.94 (0.48-1.86) | | 1.33 (0.41-4.32) | | | 0.67 (0.37-1.22) | | |
| AAEs only | | 0.32 (0.04-2.51) | | 0.74 (0.08-6.74) | | | 0.64 (0.08-5.27) | | 0.39 (0.05-3.11) | | NA | | | 0.74 (0.16-3.43) | | NA | | | 0.48 (0.11-2.17) | | |
| ACEs and AAEs | | 0.96 (0.50-1.84) | | 0.84 (0.29-2.39) | | | 0.55 (0.24-1.23) | | 1.17 (0.55-2.51) | | 0.33 (0.11-0.96) | | | 1.19 (0.59-2.39) | | 1.72 (0.51-5.76) | | | 0.69 (0.37-1.30) | | |
| **Physical, psychological, cognitive multimorbidity** | | | | | | | | | | | | | | | | | | | | | |
| **ACEs** | |  | |  | | |  | |  | |  | | |  | |  | | |  | | |
| 0 | | 1.0 Ref | | 1.0 Ref | | | 1.0 Ref | | 1.0 Ref | | 1.0 Ref | | | 1.0 Ref | | 1.0 Ref | | | 1.0 Ref | | |
| 1 | | 1.44 (0.58-3.56) | | 0.86 (0.22-3.39) | | | 1.05 (0.30-3.69) | | 1.21 (0.47-3.11) | | 1.03 (0.09-11.40) | | | 1.15 (0.52-2.53) | | 3.10 (0.38-25.28) | | | 0.97 (0.43-2.19) | | |
| ≥2 | | 1.51 (0.66-3.47) | | 1.16 (0.36-3.76) | | | 0.98 (0.31-3.14) | | 1.68 (0.73-3.84) | | 4.24 (0.58-31.21) | | | 1.12 (0.55-2.30) | | 4.59 (0.63-33.48) | | | 1.00 (0.49-2.07) | | |
| ACEs (1-indicator per increasing)^a^ | | 1.22 (1.07-1.40) | | 1.09 (0.89-1.33) | | | 1.06 (0.90-1.25) | | 1.30 (1.12-1.51) | | 1.69 (1.25-2.28) | | | 1.12 (0.99-1.27) | | 1.27 (1.03-1.57) | | | 1.15 (1.01-1.31) | | |
| **AAEs** | |  | |  | | |  | |  | |  | | |  | |  | | |  | | |
| 0 | | 1.0 Ref | | 1.0 Ref | | | 1.0 Ref | | 1.0 Ref | | 1.0 Ref | | | 1.0 Ref | | 1.0 Ref | | | 1.0 Ref | | |
| 1 | | 0.66 (0.38-1.13) | | 1.13 (0.65-1.99) | | | 1.10 (0.64-1.90) | | 0.67 (0.38-1.16) | | 0.34 (0.10-1.12) | | | 0.98 (0.65-1.48) | | 1.02 (0.49-2.13) | | | 0.78 (0.50-1.23) | | |
| ≥2 | | 1.75 (1.14-2.67) | | 0.94 (0.46-1.93) | | | 1.54 (0.93-2.57) | | 1.37 (0.81-2.32) | | 0.23 (0.03-1.73) | | | 1.69 (1.16-2.46) | | 1.70 (0.83-3.46) | | | 1.35 (0.89-2.07) | | |
| AAEs (1-indicator per increasing)^a^ | | 1.25 (1.07-1.46) | | 1.07 (0.85-1.35) | | | 1.21 (1.02-1.44) | | 1.14 (0.94-1.39) | | 0.56 (0.28-1.11) | | | 1.24 (1.09-1.42) | | 1.29 (1.00-1.66) | | | 1.14 (0.98-1.33) | | |
| **Life-course Adversity** | |  | |  | | |  | |  | |  | | |  | |  | | |  | | |
| None | | 1.0 Ref | | 1.0 Ref | | | 1.0 Ref | | 1.0 Ref | | 1.0 Ref | | | 1.0 Ref | | 1.0 Ref | | | 1.0 Ref | | |
| ACEs only | | 1.35 (0.55-3.34) | | 1.23 (0.29-5.17) | | | 1.08 (0.26-4.47) | | 1.46 (0.59-3.63) | | 3.46 (0.47-25.66) | | | 1.06 (0.47-2.43) | | 3.24 (0.44-23.78) | | | 0.99 (0.43-2.27) | | |
| AAEs only | | 0.69 (0.08-5.92) | | 1.47 (0.13-16.50) | | | 1.96 (0.18-21.77) | | 0.62 (0.07-5.31) | | NA | | | 1.10 (0.22-5.48) | | NA | | | 0.98 (0.20-4.90) | | |
| ACEs and AAEs | | 1.51 (0.59-3.84) | | 1.29 (0.30-5.50) | | | 1.40 (0.33-5.84) | | 1.36 (0.52-3.51) | | 1.04 (0.12-9.39) | | | 1.36 (0.59-3.18) | | 4.13 (0.55-31.06) | | | 1.00 (0.43-2.37) | | |
| Abbreviations: ACEs, adverse childhood experiences; AAEs, adverse adulthood experiences; HR, hazard ratio; CI, confidence interval; Ref, reference. ^a^ Continuous variable.  All subgroup analyses were adjusted for for age, sex, residential area, marital status, educational level, smoking, drinking and physical activity. The subgroup for tertiary educational level (n=68) was not reported due to insufficient sample size for meaningful analysis as per predefined criteria. | | | | | | | | | | | | | | | | | | | | | |

| **Table S9.** Sensitivity Analysis: Mutual Adjustment of Adverse Childhood and Adulthood Experiences with Multimorbidity Risk | | | | |
| --- | --- | --- | --- | --- |
| **Exposure to ACEs and AAEs** | **Physical–psychological multimorbidity** | **Physical–cognitive multimorbidity** | **Psychological–cognitive multimorbidity** | **Physical–psychological–cognitive multimorbidity** |
|  | **HR95%CI)** | **HR(95%CI)** | **HR(95%CI)** | **HR(95%CI)** |
| **ACEs (**Adjustment of AAEs**)** |  |  |  |  |
| 0 | 1.0 Ref | 1.0 Ref | 1.0 Ref | 1.0 Ref |
| 1 | 1.25 (0.91-1.72) | 0.86 (0.59-1.25) | 0.94 (0.53-1.66) | 1.26 (0.59-2.67) |
| ≥2 | 1.48 (1.11-1.97) | 0.95 (0.68-1.32) | 0.99 (0.60-1.63) | 1.41 (0.72-2.78) |
| ACEs (1-indicator per increasing)a | 1.14 (1.09-1.19) | 1.07 (1.01-1.14) | 1.13 (1.02-1.24) | 1.17 (1.05-1.31) |
| **AAEs (**Adjustment of ACEs**)** |  |  |  |  |
| 0 | 1.0 Ref | 1.0 Ref | 1.0 Ref | 1.0 Ref |
| 1 | 1.16 (1.00-1.35) | 0.91 (0.73-1.12) | 0.81 (0.58-1.14) | 0.84 (0.57-1.23) |
| ≥2 | 1.47 (1.25-1.72) | 1.24 (1.01-1.54) | 1.41 (1.02-1.94) | 1.41 (0.98-2.03) |
| AAEs (1-indicator per increasing)a | 1.16 (1.09-1.22) | 1.10 (1.02-1.19) | 1.13 (1.01-1.27) | 1.16 (1.02-1.32) |
| Abbreviations: ACEs, adverse childhood experiences; AAEs, adverse adulthood experiences; HR, hazard ratio; CI, confidence interval; Ref, reference. ^a^ Continuous variable.  Model adjusted for for age, sex, residential area, marital status, educational level, smoking, drinking and physical activity. | | | | |


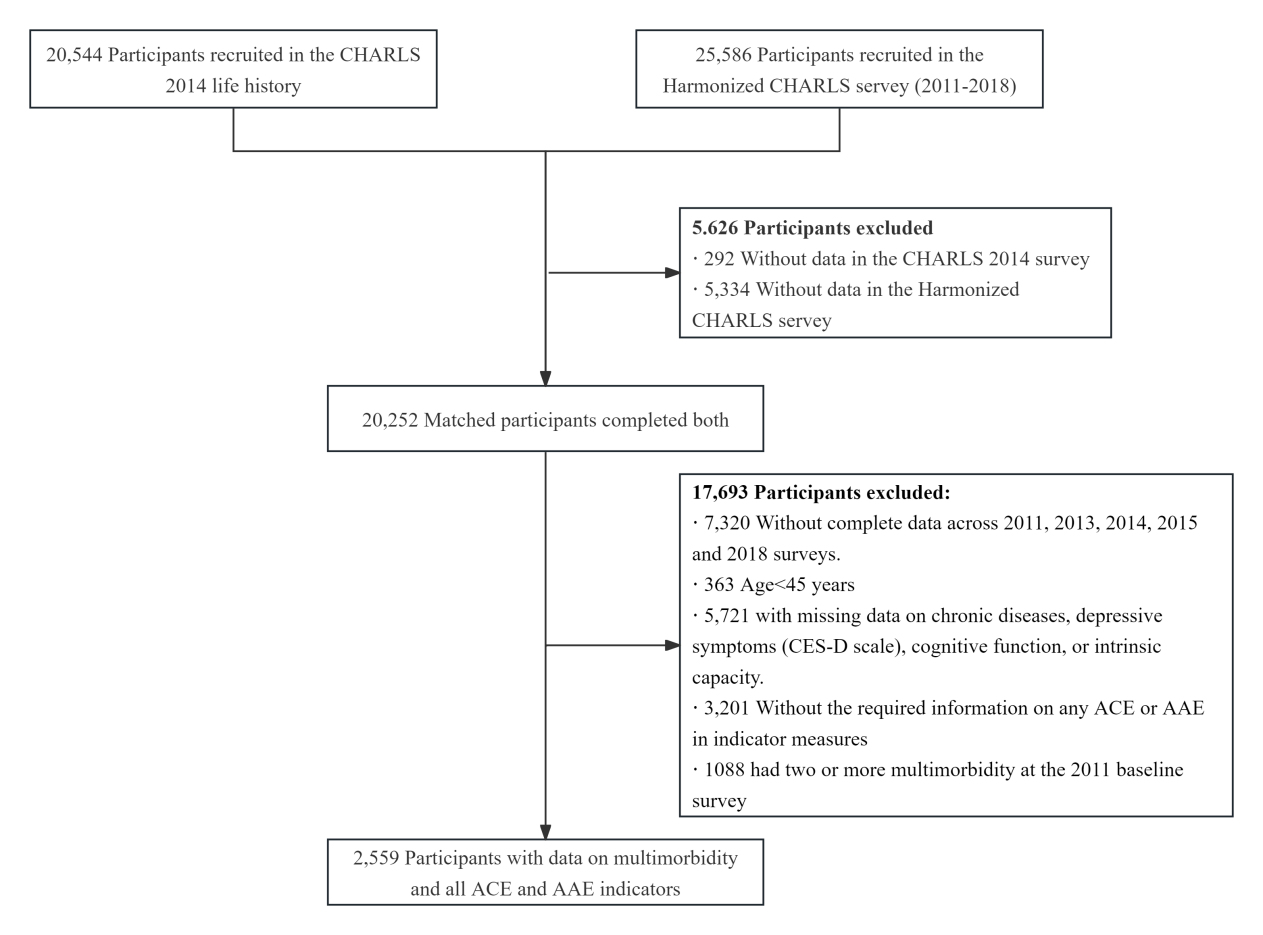


**Figure S1.** Flow diagram of study population.

Abbreviations: ACEs, adverse childhood experiences; AAEs, adverse adulthood experiences


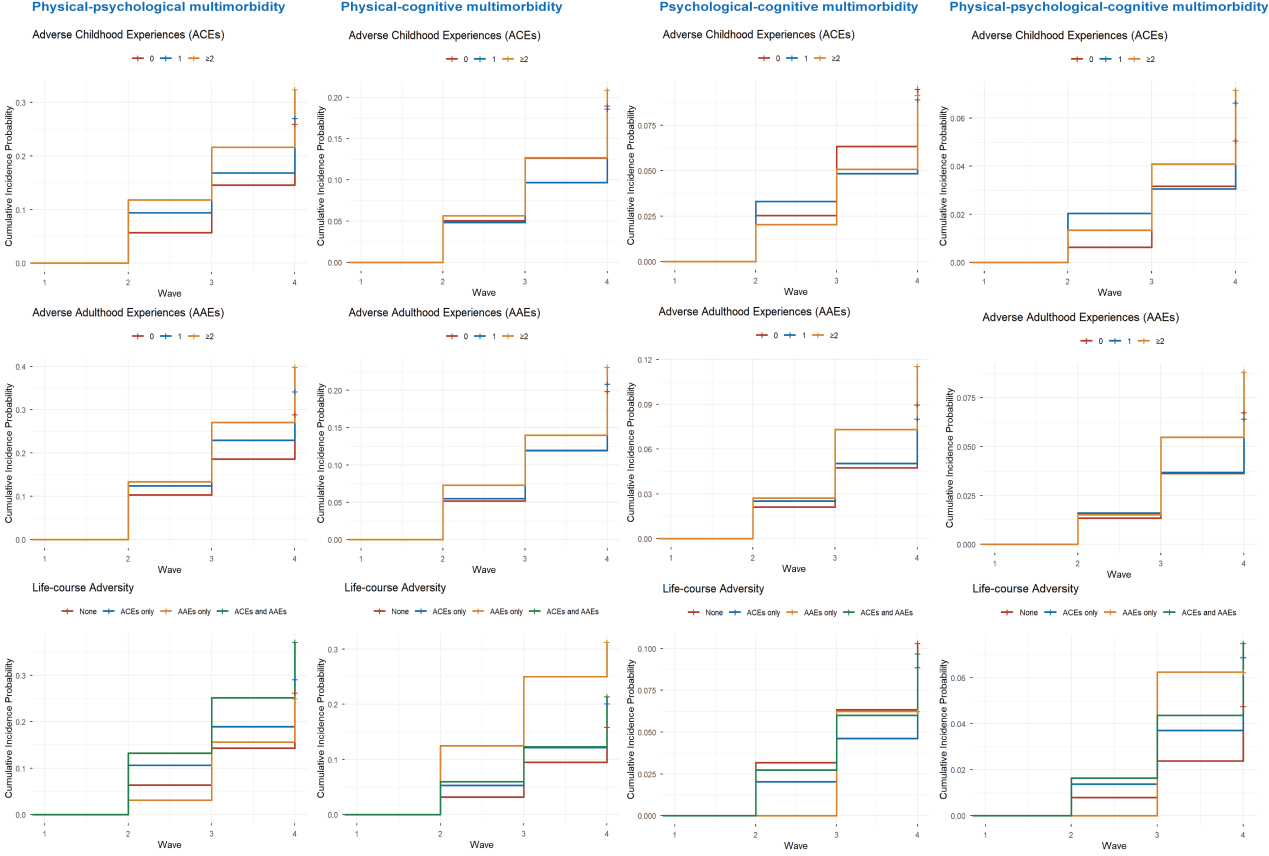


**Figure S2.** Cumulative incidence of physical, psychological, and cognitive multimorbidity according to ACEs, AAES, and life-course Adversity.

Abbreviations: ACEs, adverse childhood experiences; AAEs, adverse adulthood experiences

**
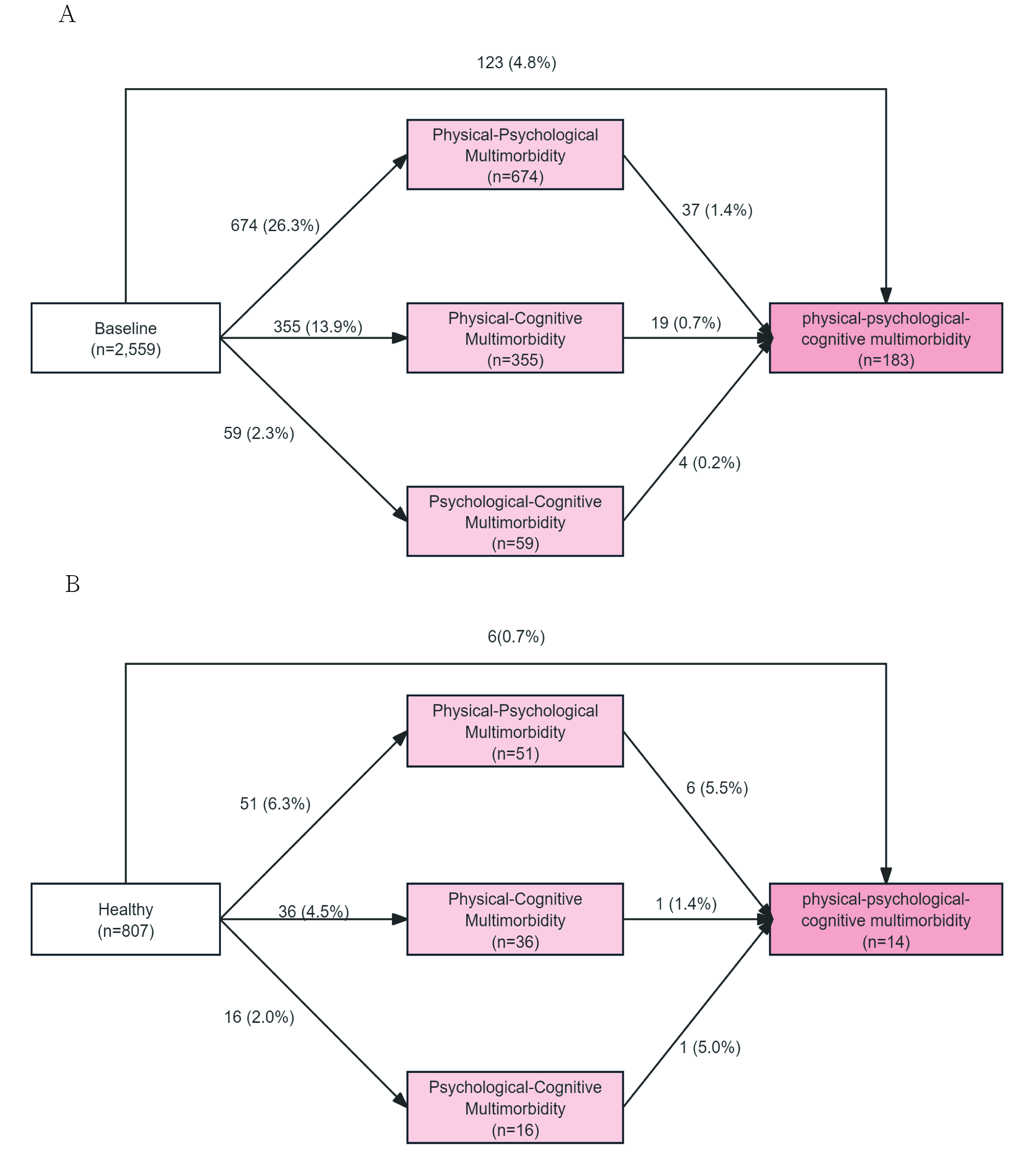
**

**Figure S3.** Multi-state models for the association of adverse childhood and adulthood experiences with transitions of physical, psychological, and cognitive multimorbidities. (A) Overall transitions in the full cohort (n = 2,559). (B) Transitions originating from the initially healthy sub-cohort (n = 807).
